# Supplementary material for: Inbreeding in Chinese Fir: Insight into the Rare Self-Fertilizing Event from a Genetic View
Source: Genes (Basel). 2022 Nov 13;13(11):2105. doi: 10.3390/genes13112105 (PMC9690749; doi:10.3390/genes13112105)
Supplement: Supplementary file 1 [file genes-13-02105-s001.zip › Figure S1.pdf]

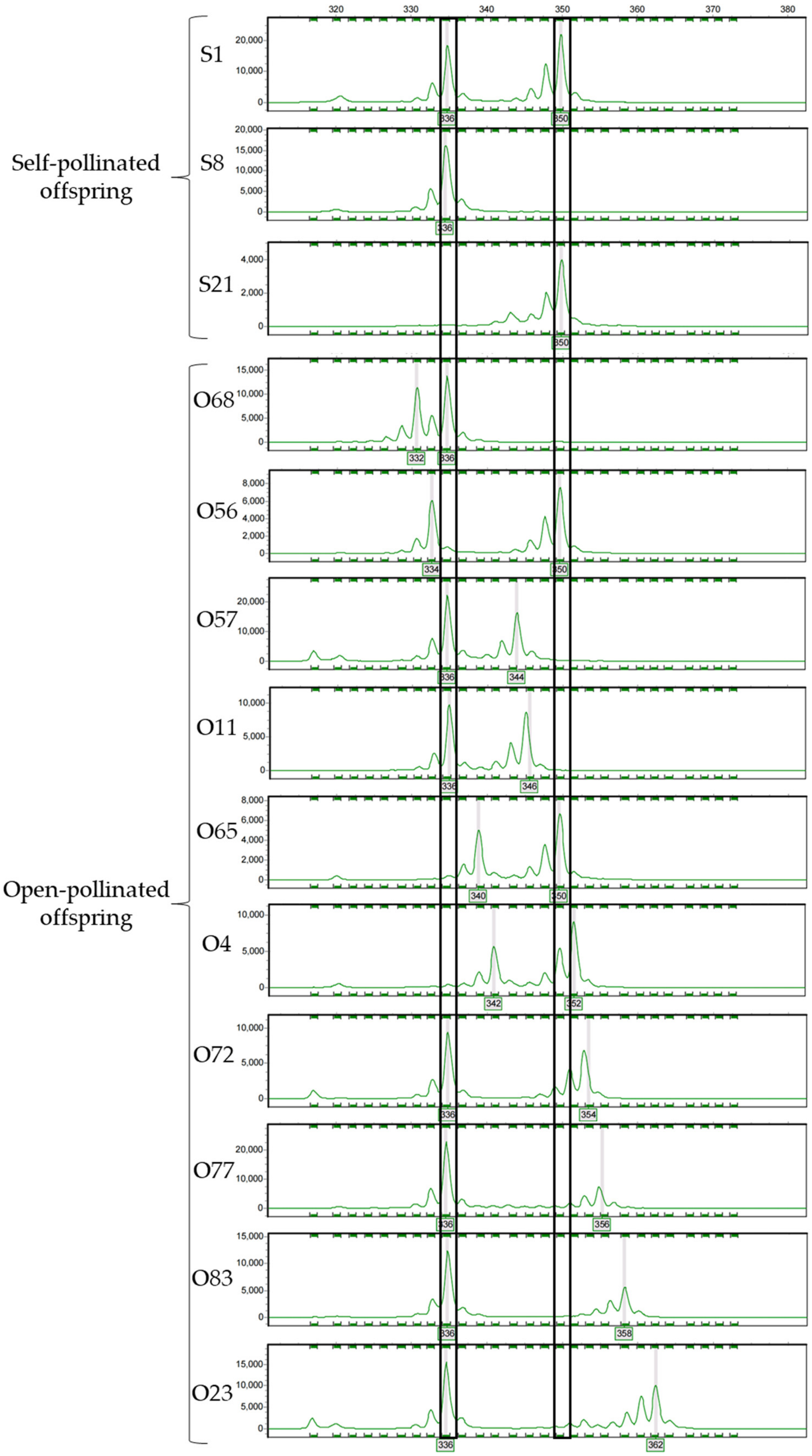

Figure S1. The fingerprint profiles of self- and open-offspring generated by primer SSR1. The fluorescence strength was shown in the Y-axis and DNA fragment sizes in base pairs was shown in the X-axis. The allele detected both in self- and open-pollinated offspring was represented by black box.
