# Supplementary material for: Inbreeding in Chinese Fir: Insight into the Rare Self-Fertilizing Event from a Genetic View
Source: Genes (Basel). 2022 Nov 13;13(11):2105. doi: 10.3390/genes13112105 (PMC9690749; doi:10.3390/genes13112105)
Supplement: Supplementary file 1 [file genes-13-02105-s001.zip › Table S1.pdf]

Table S1. Attributes of 20 microsatellite loci in this study.

Locus	Primer sequence (5′-3′)	Repeat motif	GenBank accession No	Product size range (bp)	Allele number	
					Open-pollination	Self-pollination
SSR1	F:CAATCAGCCAAGTTGTACAGGC<br>R:CATACCTTAGCAAAGCCCTCAGC	(AT)8(AG)18	AB757708<br>AB757709	332-362	14	2
SSR2	F:TGAAATTGCGTTGTACCGAAGG<br>R:TAACGAGACGAGCGACAATCTCC	(GA)13	AB749554<br>AB749555	261-289	12	2
SSR3	F:GATCCTCTGGTACTTGGTGCCC<br>R:TGCAAAGTCATGTCATCTCTGGC	(AT)9	AB749556<br>AB749557	163-179	5	1
SSR4	F:TGAATGGACTGCCACAAATTCC<br>R:TTCTTTGCAGGAAAGCCAACAAG	(AG)11	AB749550<br>AB749551	277-299	7	1
SSR5	F:CTTAAGATAGCAGCGGGAATGG<br>R:CTTGCTCGATTTCTTGCATCTGG	(CT)11	AB749562<br>AB749563	246-264	8	2
SSR7	F:AAGAGAAGAGGAGGAGGTCCAAG<br>R:CAGGAGCAGGTGCAGTAGCATTC	(AG)9	AB749570<br>AB749571	258-270	5	2
SSR8	F:ATTATCCGAGGCAGATACGCAC<br>R:CTTCTCCGTATTTGATCCATCGC	(GGA)10	AB749572<br>AB749573	332-341	4	1
SSR9	F:GAGCCGTGAAGAACGAAGGTCTC<br>R:ACGATCGGATTGTCTCAGAAACG	(GAA)12	AB749574<br>AB749575	249-267	6	2
SSR10	F:TGATCTTGGCATGTCAGTCTGG<br>R:TGTCTGTCTGCCTGCAGTTATGC	(AT)9	AB749576<br>AB749577	121-145	7	1
SSR11	F:TCATCAGCCTCAGTTTGTACTTGC<br>R：GCAATCATGGGCTCTCTGCAC	(AT)9	AB749584<br>AB749585	335-375	14	2
SSR12	F:AATGCGACTTGCAAATTTCTGG<br>R:CGAATTCCTCAATCACTTGGCTG	(AGA)10	AB749582<br>AB749583	225-246	5	1
SSR13	F:GCGGCCATTTATATCATCTTC	(GAA)9	AB749588	106-115	4	2

	R:CACGCCTGTAATTCATCTCCGTC		AB749589			
SSR14	F:GGTACTGCGAATCTTCAAATCC<br>R:TGTTCAAGAAAGGAAGCAAACGG	(TC)9	AB749580<br>AB749581	266-292	7	1
SSR15	F:TTTGGGACCTTATGGAGGTGGAG<br>R:AAACCACCAGGTTGAGAAGCAGC	(GGA)9	AB749602<br>AB749603	123-144	5	2
SSR16	F:TTTCGGCTCTCCGACTCCTTAAC<br>R:AGAATCGCGTCCAGAACACAGAG	(CT)11	AB749594<br>AB749595	118-132	5	2
SSR17	F:TCCAGGAGTCTGTGAATCCGAAG<br>R:CAGTACCAATTCAACCCAGCAGC	(CTG)9	AB749600<br>AB749601	216-222	3	2
SSR18	F:TCAAGAAGTTCCGCCATTGAGAG<br>R:CCCATGAGGATTCAGAAACATGC	(CTT)10	AB749590<br>AB749591	337-349	5	2
SSR19	F:TTAATGGTGCAAGGTGGAATTGG<br>R:TTGAATCCCACTGATCACACTGC	(GAA)10	AB749592<br>AB749593	264-270	3	1
SSR20	F:CTGTTTGTACATTGGCCTCGAC<br>R:TCACAAACCACTGTGCTGGAATG	(CTC)9	AB749596<br>AB749597	120-129	3	2
SSR21	F:GTTGGGATGTCATCAAGATTGG<br>R:TGAAAGAGGCGGAAATTGGTAGG	(TC)11	AB749598<br>AB749599	208-256	10	2
Total					132	33
Mean					6.6	1.7

F indicates forward primer; R indicates reverse primer.
